# *Aronia melanocarpa Elliot* Reduces the Activity of Angiotensin I-Converting Enzyme—*In Vitro* and *Ex Vivo* Studies

**DOI:** 10.1155/2014/739721

**Published:** 2014-06-23

**Authors:** Joanna Sikora, Marlena Broncel, Elżbieta Mikiciuk-Olasik

**Affiliations:** ^1^Department of Pharmaceutical Chemistry and Drug Analysis, Medical University of Lodz, Muszynskiego 1, 90-151 Lodz, Poland; ^2^Department of Internal Diseases and Clinical Pharmacology, Kniaziewicza 1/5, 91-347 Lodz, Poland

## Abstract

*Purpose*. The aim of the study was to analyze the effects of two-month supplementation with chokeberry preparation on the activity of angiotensin I-converting enzyme (ACE) in patients with metabolic syndrome (MS). During the *in vitro* stage of the study, we determined the concentration of chokeberry extract, which inhibited the activity of ACE by 50% (IC_50_).* Methods*. The participants (*n* = 70) were divided into three groups: I—patients with MS who received chokeberry extract supplements, II—healthy controls, and III—patients with MS treated with ACE inhibitors.* Results*. After one and two months of the experiment, a decrease in ACE activity corresponded to 25% and 30%, respectively. We documented significant positive correlations between the ACE activity and the systolic (*r* = 0.459, *P* = 0.048) and diastolic blood pressure, (*r* = 0.603, *P* = 0.005) and CRP. The IC_50_ of chokeberry extract and captopril amounted to 155.4 ± 12.1 *μ*g/mL and 0.52 ± 0.18 *μ*g/mL, respectively.* Conclusions*. Our *in vitro* study revealed that chokeberry extract is a relatively weak ACE inhibitor. However, the results of clinical observations suggest that the favorable hypotensive action of chokeberry polyphenols may be an outcome of both ACE inhibition and other pleotropic effects, for example, antioxidative effect.

## 1. Introduction

Various supplements play an important role in the prevention of cardiovascular disorders. Although the percentage of medical preparations of plant origin used in Europe is estimated at approximately 35%, they are manufactured from only 0.1% of known plant species. Therefore, new active preparations of plant origin are still being researched. Chokeberry (*Aronia melanocarpa Elliot*), from the Rosaceae family, is one of the plants displaying huge medical potential [1]. Mature chokeberry fruits and leaves represent raw food and pharmaceutical material. Despite the relatively acerb quality of its berries, the interest in chokeberry is still rising, mainly because of its health-promoting properties, but also due to its unusually easy cultivation. Chokeberry has low requirements regarding the quality of soil, is resistant to frost, and does not need spraying against pests. As a result, chokeberry fruits are free from pesticides; moreover, they have little to no heavy metal accumulation. The pharmacological properties of chokeberry are provided by a number of active compounds, including polyphenols, such as anthocyanins, flavonoids, and phenolic acids [2, 3].

Available data emphasize strong antioxidant and anti-inflammatory properties of chokeberry fruits. They were discovered to stimulate the synthesis of nitric oxide in vascular endothelium, decrease blood pressure, and reduce resistance to insulin, thus improving the glycemic profile. The use of chokeberry preparations can also result in a decreased concentration of LDL, favorable changes in plasma clotting and fibrinolytic systems, and thrombocyte activity. Due to these properties, the preparations of* Aronia melanocarpa Elliot* are postulated to be a potentially important tool in the prevention and management of many civilization-related disorders [4, 5]. While the mechanism of antioxidative action of chokeberry fruits is relatively well understood, explaining their other effects requires extensive biochemical analyses. For example, the mechanism of the hypotensive effect of chokeberry fruits has not been elucidated thus far. The question of whether the hypotensive properties result from direct inhibition of angiotensin I-converting enzyme (ACE), a key enzyme involved in regulation of blood pressure, still remains open.

The aim of the study was to analyze the effects of two-month supplementation with* Aronia melanocarpa Elliot* preparation on the activity of angiotensin I-converting enzyme (ACE) in patients with metabolic syndrome (MS). During the subsequent stage of the study we verified whether the chokeberry extract can directly inhibit the enzymatic activity of ACE* in vitro*.

## 2. Subjects and Methods

### 2.1. Reagents

Infinity ACE assay, Infinity ACE calibrators, and Infinity ACE controls were obtained from Thermo Scientific (Australia). ACE substrate for* in vitro* analysis (N-[3-(2-furyl)-acryloyl]-L-phenyl-alanylglyclglycine; FAPGG) was purchased from Sigma-Aldrich (Munich, Germany). The reagents for the preparation of 55 mmol/L Tris buffer (pH 8.2) were obtained from Polish Chemical Reagents (Gliwice, Poland). Captopril (Jelfa, SA Jelenia Góra, Poland) was used as a standard competitive inhibitor of angiotensin-converting enzyme (ACE).* Aronia melanocarpa Elliot* extract (Aronox) was purchased from Agropharm SA (Poland). Aronox (100 mg capsules) is a standardized dietary supplement. According to the manufacturer, the extract contained ca. 60 mg of total polyphenols, including a minimum of 20 mg of anthocyanins: 3-O-cyanidin-galactoside (64.5%), 3-O-cyanidin-arabinoside (28.9%), 3-O-cyanidin-xyloside (4.2%), and 3-O-cyanidin-glucoside (2.4%).

## 3. Patients and Study Design

The patients were recruited from the Department of Internal Diseases and Clinical Pharmacology, Medical University of Lodz. The protocol of the study was approved by the Bioethics Commission of that institution (no. 241/06/KB). All subjects gave their written informed consent prior to participating in the study.

The study included 70 subjects subdivided into three groups. The study group (I) (*n* = 25) consisted of nontreated patients with metabolic syndrome (14 women, 11 men, 50–69 years old). The reference groups (*n* = 45; 28 women, 17 men, 55–71 years old) consisted of 20 healthy volunteers (II) and 25 patients with metabolic syndrome (III), who have received hypolipidemic and antihypertensive therapy. Participants were instructed not to modify their usual food intake and physical activity during the study. Additionally, ingestion of products containing black chokeberry (juices, jams, fresh, or frozen fruits) was prohibited. The detailed inclusion and exclusion criteria were previously described [6, 7]. Subjects with MS from group I were treated with capsules containing 100 mg of* Aronia melanocarpa Elliot* extract three times daily during the two-month study period.

Three control visits were scheduled for the subjects from group I, prior to the initiation of treatment, after one month of therapy, and after two months of therapy. Patients from the reference groups (II and III) were examined once. During the visits, the subjects underwent clinical examination, measurement of body weight and waist circumference, and venous blood sampling in order to evaluate the studied parameters and the safety laboratory parameters, lipidogram, and glucose levels. Arterial blood pressure was measured three times in the right arm, with the patient in a sitting position. The diagnosis of hypertension was confirmed if the average systolic or diastolic pressure was >140 mmHg or >90 mmHg, respectively, or if use of prescription antihypertensive medication was reported. Blood samples were taken, after an overnight fast, between 8:00 and 9:00 a.m., in order to avoid circadian fluctuations. The samples were immediately coded, so that the person performing the laboratory assay was blinded to the subject's identity and the study sequence. Compliance was assessed during each visit by tablet counts and was considered satisfactory when the number of tablets taken by the patient ranged from 90% to 100%.

## 4. Sample Preparation

Blood for ACE-activity analysis was collected into Vacuette coagulation tubes (Greiner Bio-One, Austria) containing 3.2% buffered sodium citrate. Plasma for analysis was obtained by centrifuging the blood (2500 ×g, 20 min, 4°C). Plasma was stored at −70°C until the measurements were performed. The safety laboratory parameters, lipidogram, CRP, and glucose levels were determined by routinely used methods at the Dr. W. Bieganski Voivodeship Specialist Hospital in Lodz.

## 5. ACE Activity

The ACE activity was measured with commercially available Infinity ACE assay. The assay is linear between 1 and 120 U/L of ACE activity. For higher values of ACE activity, plasma prior analysis was appropriately diluted. The Infinity ACE is widely used for monitoring the effects of ACE inhibitors in the treatment of hypertension and heart failure. ACE activity measurement is also used to aid the differential diagnosis of clinically active pulmonary sarcoidosis and for monitoring the effectiveness of steroid therapy. Analyses were carried out with assay protocol on multimode microplate Synergy H1 reader (Bio-Tek Instruments, Inc., USA).

The* in vitro* evaluation of ACE activity was performed based on the continuous measurement of absorbance, using a Cecil CE2021 spectrophotometer (Cecil, London, England). Registration and evaluation of the results were performed using the software for kinetic studies (DATA STREAM CE3000 5.0). In this method, the direct substrate N-[3-(2-furyl)-acryloyl]-L-phenyl-alanylglyclglycine (FAPGG) is hydrolyzed by plasma ACE to FAP and glycylglycine. The hydrolysis of FAPPG by ACE results in a decrease in absorbance at 340 nm.

During* in vitro* study, 40 *μ*L of plasma was incubated (3 min, 37°C) with 10 *μ*L of chokeberry extract dissolved in distilled water (1–300 *μ*g/mL), or 10 *μ*L of water solution of captopril (0.0022–22 *μ*g/mL), or 10 *μ*L of distilled water (control sample). After incubation, 400 *μ*L of FAPGG substrate (0.80 mmol/L) was added to a cuvette with plasma and a 10-minute continuous recording of ACE activity was initiated. The activity of ACE (U/L) was calculated with the aid of Infinity ACE calibrators. The reliability of the method was verified with Infinity ACE control plasma: plasma N (24–48 U/L) and plasma E (53–95 U/L), the ACE activity of which amounted to 32.0 ± 4.5 U/L (*n* = 5) and 70.4 ± 13.7 U/L (*n* = 5), respectively.

The percentage of ACE inhibition was calculated according to the equation ((*A*_0_ − *A*_IC_)∗100%)/*A*_0_, where *A*_0_ − ACE activity in the control sample, expressed as the rate of the enzymatic reaction [*A*/min], *A*_IC_ − ACE activity in the sample with the extract of chokeberry, or captopril expressed as the rate of the enzymatic reaction [*A*/min]. The curve of percent inhibition of enzyme activity depending on extract concentration in the sample was prepared to determine the IC_50_ value. The IC_50_ value was calculated according to the equation *y* = *a*∗ln⁡(*x*) + *b* (captopril) and *y* = *a*∗*x* + *b* (chokeberry extract).

## 6. Data Analysis

All values are expressed as mean ± SD. Statistical tests were performed using a commercial software package (Statistica 8.0). The Kolmogorov-Smirnov test was used to determine whether the continuous data were normally distributed. For within-group comparison, the data indicating abnormal distributions were evaluated using the Mann-Whitney test or the Wilcoxon signed-rank test for paired-sample, and data indicating normal distributions were evaluated by the *t*-test or the paired *t*-test. Correlations were determined by Pearson's correlation analysis. *P* > 0.05 was considered statistically significant.

## 7. Results

The characteristics of the three groups of participants included in the study are presented in Table 1. No significant adverse events were recorded, and all patients completed the study. All laboratory safety measurements remained within reference limits. Group I comprised of previously untreated patients with metabolic syndrome. Compared to the healthy controls and patients from group III, they showed significantly higher mean values of total cholesterol and LDL concentrations, BMI, waist circumference, and blood pressure (SBP, DBP), and their mean concentration of HDL was significantly lower compared to the control group. Group III included patients with MS and concomitant arterial hypertension, who were treated with ACE inhibitors for at least three months. Additionally, various protocols of hypolipidemic treatment were used for patients from this group. A significant, several percent, decrease in the concentrations of TC and LDL and in the level of SBP was documented after two months of chokeberry preparation administration. However, despite significant decrease in LDL and SBP, the values of these parameters still remained significantly higher than in the controls (II).

Mean ACE activity in the three studied groups and the effects of one- and two-month supplementation with chokeberry extract on this parameter are summarized in Table 2. Patients from group I showed significantly higher ACE activity than the controls (group II) and individuals from group III. Although a significant decrease in the ACE activity was observed after both one and two months of supplementation with Aronox, this parameter still remained significantly higher as compared to both reference groups.

We calculated coefficients of correlation between the ACE activity and selected parameters: systolic and diastolic blood pressure, body mass index (BMI), and C-reactive protein (CRP) of subjects from all three study groups (Table 3). Moreover, relations between ACE activity and the concentrations of total cholesterol, LDL, and HDL were analyzed, but none of them proved statistically significant (data not shown).

**Figure 1:**
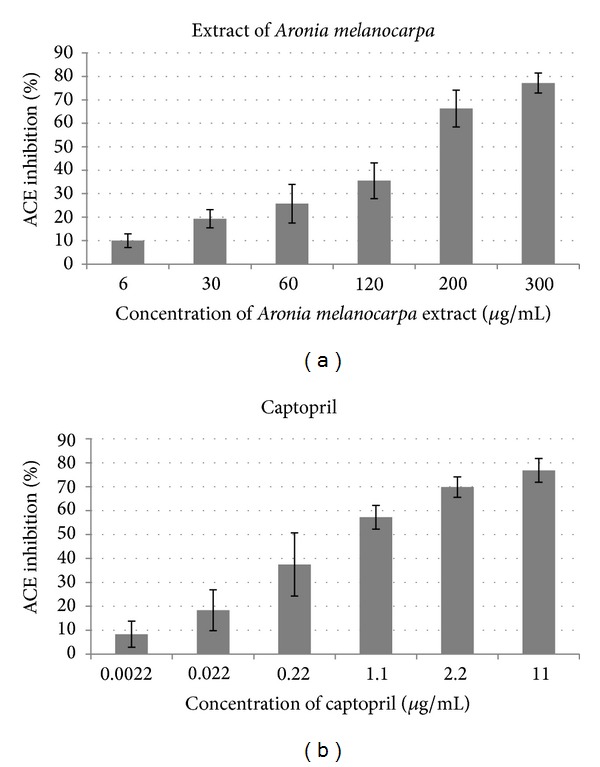
Inhibition of angiotensin I-converting enzyme (ACE) activity by (a) polyphenol-rich extracts of* Aronia melanocarpa Elliot *(*n* = 6) and (b) captopril (standard for ACE-inhibitor; *n* = 5). The results are expressed as the means ± standard deviation of percentage of ACE inhibition.

The percent of ACE inhibition obtained in* in vitro* tests during incubation of plasma from healthy volunteers with various concentrations of captopril and chokeberry extract is presented in Figures 1(a) and 1(b). The concentrations of captopril and chokeberry extract that inhibited the activity of ACE by 50% (IC_50_) were evaluated. Mean IC_50_ values for the solutions of standard ACE inhibitor (captopril) and chokeberry extract amounted to 0.52 ± 0.18 *μ*g/mL and 155.4 ± 12.1 *μ*g/mL, respectively.

## 8. Discussion

The increased prevalence of obesity-related disorders stimulated research on new methods of preventing and treating such conditions as arterial hypertension, hyperlipidemia, and diabetes mellitus. The results of many epidemiological studies suggest that daily ingestion of plant polyphenols can reduce the risk of atherosclerosis through its favorable effect on lipid profile and blood concentration of glucose and the reduction of blood pressure and predisposition to thrombosis [8]. Previous studies have shown positive influence of supplementation with chokeberry extract on the concentration of lipids, blood pressure, and the levels of endothelin-1 and the markers of oxidative stress and hemostasis [6, 7].

Our study confirmed previously reported positive effects of supplementation with chokeberry preparation on lipid metabolism and blood pressure [4, 9]. During enrollment, patients with MS showed significantly higher concentrations of total cholesterol and LDL and significantly lower concentrations of HDL than the healthy controls (group II); however, they did not differ significantly from individuals from group III, that is, patients with MS who received hypolipidemic treatment (TC *P* = 0.980, LDL *P* = 0.540, and HDL *P* = 0.192). The type of dyslipidemia characterized by concomitant increase in LDL and decrease in HLD concentration is considered to be strongly atherogenic. We observed a significant decrease in TC and LDL concentrations in our patients after both one and two months of receiving chokeberry extract. In contrast, the level of HDL remained unchanged and was still significantly lower than in healthy controls. We did not find any relation between the levels of total cholesterol, LDL, and HDL, and the plasma activity of ACE in any of the three studied groups. Similar association was previously documented in an animal model of experimentally induced hypercholesterolemia. Rabbits with induced hypercholesterolemia showed a 15% increase in the serum activity of ACE. This finding may point to potential association between renin-angiotensin system and lipid metabolism [10].

Angiotensin I-converting enzyme (ACE, EC3.4.15.1) is a zinc metallocarboxypeptidase, mostly commonly found in vascular endothelial cells and neuroepithelial cells. ACE plays a significant physiological role in regulating blood pressure in the renin-angiotensin system, because it can transform angiotensin from an inactive decapeptide (angiotensin-I) to a strong vasoconstrictor octapeptide (angiotensin-II). In addition, ACE also inactivates bradykinin, a potent vasodilatory peptide [11, 12].

At present, the therapeutic protocols of arterial hypertension include a number of synthetic inhibitors of ACE, such as captopril, lisinopril, or enalapril. MS constitutes absolute indication for pharmacotherapy in patients with arterial hypertension and relative indication for treatment in individuals with high regular pressure. ACE inhibitors are frequently prescribed and have an established position in these indications, as the benefits associated with their use usually outweigh the potential risks. However, particular care should be taken if the inhibitors of ACE are administered to women at reproductive age, who do not use contraceptives or plan on getting pregnant. Moreover, due to their teratogenicity, ACE inhibitors are contraindicated in pregnant patients. Furthermore, they can penetrate into human breast milk and thus should be administered with particular care to breastfeeding women.

All the above-mentioned limitations constitute one of the reasons behind the growing interest of physicians and researchers in alternative, plant-derived ACE inhibitors. For example, a large number of ACE inhibitory peptides have been isolated and identified from enzymatic hydrolyzates and fermentation products of milk, fish, soybean, peanut, and canola proteins [11–13]. Lots of studies have demonstrated that certain flavonoids originating from berries, other fruits, or vegetables can have an inhibitory effect on ACE activity [14]. In contrast, the number of studies dealing with the effect of chokeberry polyphenols on the activity of ACE is sparse. Naruszewicz et al. [15] were one of the first researchers who showed that administration of chokeberry flavonoids not only lowers blood pressure, but also decreases the activity of ACE. In a double-blind, placebo-controlled, parallel trial, an inhibition of ACE activity by about 33% was documented in 10 individuals receiving chokeberry extract. It is noteworthy that these changes were observed in patients with elevated baseline values of the studied parameters, namely, with systolic and diastolic pressure exceeding 130 mmHg and 90 mmHg, respectively, and ACE activity above 50 U/L [15].

The baseline ACE activity in our patients with MS was significantly higher than in the healthy controls (group II, *P* < 0.001) or individuals with MS treated with ACE inhibitors (group III, *P* < 0.001). Mean baseline levels of SBP and DBP in patients from groups I and III were significantly higher than in healthy controls (Table 2). After one and two months of supplementation with chokeberry extract, we observed a favorable, significant decrease in SBP, as well as a nonsignificant decrease in DBP; nevertheless, the values of these parameters still remained significantly higher than in healthy controls. Significant positive correlations between the ACE activity and the levels of SBP (*r* = 0.459, *P* = 0.048) and DBP (*r* = 0.603, *P* = 0.005) were documented in patients with MS who did not receive any pharmacotherapy (group I). Noticeably, similar associations were not observed after either one or two months of administering chokeberry extract to individuals from any of the two reference groups (groups II and III; Table 3). Also, significant correlations between ACE activity and the CRP levels documented for individuals with MS, at baseline (*r* = 0.492, *P* = 0.023) and following one month of supplementation (*r* = 0.507, *P* = 0.027), as well as the correlation between ACE activity and BMI (*r* = 0.461, *P* = 0.030) observed after two months of chokeberry extract administration, are noteworthy. These correlations may indirectly confirm that arterial hypertension is associated with inflammation and obesity in patients with MS.

Our study confirmed previous observations that the effects of chokeberry extract on ACE are the strongest in patients with higher activity of this enzyme and with higher levels of arterial pressure [15]. After one and two months of administering the chokeberry preparation, an average decrease in ACE activity corresponded to 25% (range: 8–53%) and 30% (range: 4–80%), respectively. It is noteworthy that the inhibition was the most pronounced in the case of extremely high activities of this enzyme. A strong effect of polyphenols on the activity of ACE was further confirmed by the fact that after one and two months of the supplementation, a decrease in this parameter was observed in 19 and 18 subjects, respectively, and its increase above the reference (8–52 U/L) was documented in only three patients, solely after two months of the treatment.

Guerrero et al. [14] performed* in vitro* testing of the effect of 17 flavonoids from five different structural groups on the activity of ACE. Subsequently, they determined the structure-activity relationships (SAR) for the most active compounds. They discovered that some flavonoids can act as ACE inhibitors* in vivo* at their physiological concentrations (100 *μ*M). Balasuriya and Rupasinghe [16] analyzed the effect of polyphenol-rich apple peel extract on the activity of ACE isolated from a rabbit lung. The values of IC_50_ were determined from a dozen or so flavonoids contained in the extract. Some of these flavonoids, for example, cyaniding 3-O-glucoside (IC_50_ = 174 *μ*M), cyaniding 3-O-galactoside (IC_50_ = 206 *μ*M), epicatechin (IC_50_ = 70 *μ*M), quercetin-3-O-glucoside (IC_50_ = 151 *μ*M), quercetin-3-O-galactoside (IC_50_ = 180 *μ*M), and quercetin-3-O-rutinoside (IC_50_ = 90 *μ*M), are also present in chokeberries [16].

According to the manufacturer of the chokeberry preparation used in our* in vivo* and* in vitro* experiments, one capsule with 100 mg of* Aronia melanocarpa Elliot* extract contains ca. 60 mg of total polyphenols, including a minimum of 20 mg of anthocyanins: 3-O-cyanidin-galactoside (64.5%), 3-O-cyanidin-arabinoside (28.9%), 3-O-cyanidin-xyloside (4.2%), and 3-O-cyanidin glucoside (2.4%). The chokeberry fruits are considered the richest source of these four anthocyanins, differing from one another solely with respect to their sugar residues. However, according to the available data, the type of sugar included in anthocyanin aglycone does not modulate its inhibitory activity against ACE [17, 18]. This was also confirmed during* in vitro* and cell culture studies, which showed that this enzyme can be inhibited by both anthocyanins and anthocyanin-rich plant extracts. The inhibitory activity against ACE is probably determined by hydrogen bonds formed between the active center of the enzyme and the hydroxyl groups of cyanidin in positions C-3, C-5, C-7, and C-40, as well as by the planar structure of the cyanidin molecules [14].

Hellström et al. [19] analyzed the* in vivo* effect of chokeberry juice and polyphenols on arterial blood pressure in spontaneously hypertensive rats. They revealed that both the juice and isolated chokeberry polyphenols exert a hypotensive effect, which is strongest three hours after their administration. Moreover, the* in vitro* effect of chokeberry juice and isolated polyphenols on ACE activity was examined, with hippuryl-L-histidyl-L-leucine (HHL) as a substrate. This experiment showed that chokeberry juice is a very weak inhibitor of ACE* in vitro*; its effect proved to be approximately 3∗10^6^ weaker than that of captopril (IC_50_ = 4.5 mg dry matter/mL versus IC_50_ = 1.3∗10^−6^ mg/mL for captopril).

We did not document as dramatic difference: chokeberry extract inhibit the activity of ACE only approximately 300-fold weaker than captopril. The mean value of IC_50_ for polyphenol-rich chokeberry extract, documented in our study of serum from healthy volunteers, amounted to 155.4 ± 12.1 *μ*g/mL (*n* = 6) and was relatively high compared to the IC_50_ for captopril (*n* = 5), amounting to 0.52 + 0.18 *μ*g/mL. This points to relatively weak inhibition of ACE by chokeberry extract. However, the results of our clinical observations and* in vitro* experiments, as well as correlations documented in our study, suggest that the favorable hypotensive action of chokeberry polyphenols may be an outcome of both ACE inhibition and other pleotropic effects, for example, antioxidative effect.

In view of the increasing prevalence of MS, obesity, and hypertension among younger individuals, chokeberry extract can constitute an interesting alternative for conventional pharmacotherapy in women at the reproductive age who do not use contraceptives or plan on getting pregnant.

We are aware that our study had some limitations. The small number of subjects and the design of the study (a placebo-treated group was not included) both constitute potential limiting factors. However, this experiment may be treated as a pilot study.

## Figures and Tables

**Table 1:** Characteristics of control group and patients with metabolic syndrome (mean ± SD) at baseline and after 1 (1 M) and 2 months (2 M) of *Aronia melanocarpa Elliot* extract supplementation: TC-total cholesterol, LDL-low-density lipoprotein cholesterol, HDL-high-density lipoprotein cholesterol, TG-triglyceride, BMI-body mass index, SBP-systolic blood pressure, DBP-diastolic blood pressure, CRP-C-reactive protein, NE-not evaluated, and NS-not statistically significant.

Parameters	Patients with MS (group I)			Control (group II) (*n* = 20)	Control group MS (group III) (*n* = 25)
	At baseline (*n* = 23)	After 1 M (*n* = 23)	After 2 M (*n* = 23)		
TC (mg/dL)	237.6 ± 36.9^*P*=0.002^	220.5 ± 32^NS/∗∗^	216.7 ± 33.7^NS/∗∗^	206.6 ± 32.3	224.4 ± 45.2^NS^
LDL (mg/dL)	151.7 ± 36.1^*P*<0.001^	142.7 ± 33.3^*P*=0.008/∗∗^	139.2 ± 30.9^*P*=0.013/∗∗^	122.9 ± 28.7	135.6 ± 40.1^NS^
HDL (mg/dL)	49.9 ± 11.5^*P*=0.002^	51.5 ± 13.7^*P*=0.008^	44.1 ± 12.1^*P*=0.003^	64.8 ± 16.7	59.2 ± 19.9^NS^
BMI (kg/m^2^)	30.9 ± 3.7^*P*=0.002^	30.0 ± 3.5^*P*=0.001^	30.4 ± 3.7^*P*<0.001^	23.0 ± 1.5	29.2 ± 2.8^*P*=0.005^
Waist circumference (cm)	95.0 ± 9.4^*P*<0.001^	93.4 ± 8.8^*P*=0.002^	93.7 ± 8.5^*P*=0.001^	84.6 ± 8.5	97.2 ± 8.8^*P*<0.001^
SBP mmHg	136.8 ± 10.9^*P*<0.001^	130.0 ± 9.0^*P*=0.005/∗∗^	126.3 ± 11.6^*P*=0.014/∗∗^	121.3 ± 10.2	149.0 ± 20.4^*P*=0.02^
DBP mmHg	86.8 ± 16.0^*P*=0.002^	83.1 ± 8.2^*P*<0.001^	80.5 ± 7.9^*P*=0.005^	73.5 ± 7.3	84.0 ± 7.6^*P*<0.001^
CRP mg/dL	3.1 ± 2.3	2.9 ± 2.6^NS^	2.5 ± 1.7^NS^	NE	NE

**P < 0.05 versus baseline; *P* = values versus control healthy group (group II).

**Table 2:** Values of ACE activity of control groups (II and III) and patients with metabolic syndrome at baseline and after 1 (1 M) and 2 months (2 M) of *Aronia melanocarpa Elliot* extract supplementation: MIN–MAX: minimum–maximum values. The reference interval is 8–52 U/L (37°C).

ACE activity U/L	Patients with MS (Group I)			Control (group II) (*n* = 20)	Control group MS (group III) (*n* = 25)
	At baseline (*n* = 23)	After 1 M (*n* = 23)	After 2 M (*n* = 23)		
mean ± SD	87.0 ± 42.1^*P*<0.001^	64.6 ± 25.4^*P*<0.001/∗∗∗^	61.0 ± 34.1^*P*=0.001/∗∗∗^	31.3 ± 11.6	36.6 ± 16.53^NS^
MIN–MAX	20.3–165.5	29.9–135.5	11.0–147.0	8.8–60.9	10.31–73.0

***P < 0.001 versus baseline; *P* = values versus control healthy group (group II); NS-not statistically significant.

**Table 3:** The correlation coefficients and *P* values for correlation of ACE activity and blood pressure: SBP-systolic blood pressure, DBP-diastolic blood pressure, BMI-body mass index, CRP-C-reactive protein in control group and patients with metabolic syndrome at baseline and after 1 (1 M) and 2 months (2 M) of *Aronia melanocarpa Elliot* extract supplementation; NE-not evaluated.

	Patients with MS (group I) (*n* = 23)			Control (group II) (*n* = 20)	Control group MS (group III) (*n* = 25)
	Baseline	1 M	2 M		
	ACE activity	ACE activity	ACE activity	ACE activity	ACE activity

SBP	0.459; *P* = 0.048	0.315; *P* = 0.176	−0.229; *P* = 0.329	−0.034; *P* = 0.889	0.333; *P* = 0.104
DBP	0.603; *P* = 0.005	0.350; *P* = 0.130	0.3712; *P* = 0.106	0.298; *P* = 0.216	0.267; *P* = 0.196
BMI	0.363; *P* = 0.097	0.296; *P* = 0.296	0.461; *P* = 0.030	0.034; *P* = 0.877	0.388; *P* = 0.137
CRP	0.492; *P* = 0.023	0.507; *P* = 0.027	−0.207; *P* = 0.393	NE	NE
